# Endoscopic Excision of Transsellar Transsphenoidal Meningoencephalocele Utilizing the Slip‐Knot Technique

**DOI:** 10.1002/oto2.70110

**Published:** 2025-04-21

**Authors:** Chin‐Nung Liu, Shih‐Hung Yang, Ting‐Hua Yang, Chih‐Feng Lin

**Affiliations:** ^1^ Department of Otolaryngology, Head and Neck Surgery National Taiwan University Hospital Taipei Taiwan; ^2^ Department of Surgery Division of Neurosurgery, National Taiwan University Hospital Taipei Taiwan

**Keywords:** endonasal endoscopic approach, meningoencephalocele, slip‐knot

True transsphenoidal meningoencephalocele occurs when the brain and meninges herniate through a defect in the skull and dura through sphenoid sinus into sphenoid sinus, nasopharynx, and nasal cavity.

The condition can be diagnosed from infancy to late adulthood depending on presenting symptoms. Meningoencephalocele may cause mass effect and compress adjacent structures, leading to visual symptoms, visual field defects, decreased visual acuity, or endocrine abnormalities.

Surgical intervention is warranted in patients with symptoms such as respiratory distress, cerebrospinal fluid (CSF) leaks, meningitis, or vision loss. Traditionally, surgery is performed using the transpalatal or transcranial approach, which carried high risks of mortality and complications.^1^ However, with advances in endoscopic endonasal approach, the transsphenoidal route under endoscopy has emerged as a viable alternative.^2^

The purpose of this manuscript is to report a case of true transsphenoidal meningoencephalocele managed with endoscopic excision utilizing slip‐knot technique.

## Case Report

A 21‐year‐old male presented to our pediatric clinic for short stature. Physical examination revealed a high‐pitched voice, Tanner stage II pubic hair, and small testicular volume. Blood test indicated hypopituitarism involving the somatotropic and gonadotropic axes, whereas visual test indicated bilateral temporal vision defect.

**Figure 1:**
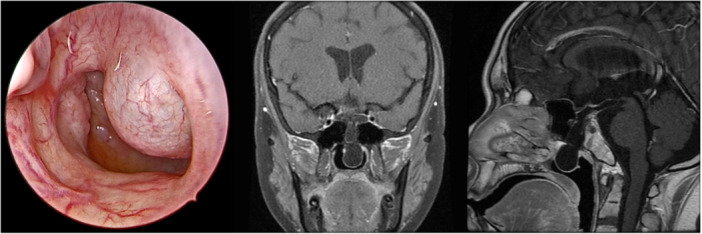
Endoscope and magnetic resonance imaging images reveal the meningoencephalocele protruding through sellar floor and sphenoid sinus into posterior nasal cavity.

Endoscope and radiology evaluations identified a fluid sac penetrating through a defect in the sphenoid and sellar floor into posterior nasal cavity and nasopharynx (Figure 1).

**Figure 2:**
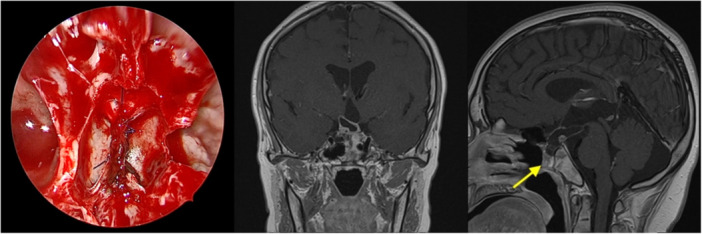
Stalk of the ligated herniated sac and postoperative magnetic resonance imaging images indicated the disappearance of the mass in the posterior nasal cavity. The arrow indicates the nasoseptal flap.

The patient underwent meningoencephalocele excision via an endoscopic endonasal approach. A right‐sided nasoseptal flap was created. Posterior septum, the anterior sphenoid sinus wall, and rostrum were removed to widen the exposure. Mucosa adherent to the sac was partially dissected. The stalk of the sac was exposed and freed from the surrounding tissue, and a mild CSF leak was observed. Bipolar cautery was used to shrink the stalk. A 5‐0 Prolene suture was applied using the slip‐knot technique at the sellar‐floor level. The stalk was transected with microscissors, and the nasal mucosa–covered sac was removed (Figure 2). The defect was reconstructed using nasoseptal flap, Gelfoam (Pfizer), and Tisseel® (Baxter) (Supplemental Video S1, available online).

Postoperative period was uneventful. There were no CSF leak or intracranial hypotension. His visual function remained stable. Although the patient reported nocturia, his urine osmolality was within normal limits. A postoperative magnetic resonance imaging conducted 3 months later confirmed that the nasoseptal flap had healed well and that there was no meningoencephalocele recurrence (Figure 2).

This case report was exempted by the Institutional Review Board of National Taiwan University Hospital (registration number: 202407227W).

## Discussion

Surgical management of true transsphenoidal meningoencephalocele is challenging due to its proximity to critical structures such as the pituitary gland, optic nerves, and blood vessels. Historically, transpalatal and transcranial approaches were commonly employed.^1^ Generally, the contents of the meningoencephalocele are considered nonfunctional and are reduced by bipolar cautery to the level of skull base defect. The surrounding mucosa is dissected to mobilize the dural sac, which is pushed superiorly into the cranium, and the defect is either closed off or reconstructed.^2^, ^3^

However, the traditional transcranial and transpalatal approaches are limited by a restricted operative field, and traction injury to adjacent structure during dissection may cause further complications such as CSF rhinorrhea. Recent advancements in endoscopic surgery have enabled the surgical resection and reconstruction of meningoencephalocele through the endoscopic endonasal approach.^2^

In our case, the sac was too large to be repositioned superiorly and too deep intracranially such that only partial resection was possible. To minimize the risk of postoperative CSF leakage, surgical ligation with 5‐0 Prolene sutures in addition to bipolar cautery to secure the transection site and prevent leakage at sellar floor.

The slip‐knot technique, inspired by a knot used for tying fishing lines, was first proposed by Japanese neurosurgeons and has since been used for deep sutures in endoscopic endonasal surgeries (Supplemental Video S1, available online).^4^

In previous reports, defects were often reconstructed using cranial or rib bone, titanium mesh, fat, fascia lata, or fibrin glue.^5^ In the past decade, advancements in endoscopic skull base surgery have led to the use of nasoseptal flap as the workhorse flap for reconstruction. In patients with small defects and low rate of CSF leakage, combining the nasal septal flap with packing may provide adequate structure support to the defect and reduce the risk of foreign body reaction.

## Author Contributions

**Chin‐Nung Liu**, acquired the data and drafted the manuscript; **Shih‐Hung Yang**, acquired the data and revised the manuscript; **Ting‐Hua Yang**, revised the manuscript; **Chih‐Feng Lin**, designed the study and approved the manuscript.

## Disclosures

### Competing interests

The authors declare no conflicts of interest.

### Funding source

The authors did not receive funding for this case report.

## Supporting information

oto open revised.

Video 1. Clinical history, surgical video, postoperative follow‐up, and demonstration of the slip‐knot technique.
