# Supplementary material for: Endoscopic Excision of Transsellar Transsphenoidal Meningoencephalocele Utilizing the Slip‐Knot Technique
Source: OTO Open. 2025 Apr 21;9(2):e70110. doi: 10.1002/oto2.70110 (PMC12010755; doi:10.1002/oto2.70110)
Supplement: Supplementary file 2 — Video 1. Clinical history, surgical video, postoperative follow‐up, and demonstration of the slip‐knot technique. [file OTO2-9-e70110-s002.docx]

Video 1. Clinical history, surgical video, post operative follow up and demonstration of the slip-knot technique.
